# Self-help and help-seeking for communication disability in Ghana: implications for the development of communication disability rehabilitation services

**DOI:** 10.1186/s12992-017-0317-6

**Published:** 2017-12-29

**Authors:** Karen Wylie, Lindy McAllister, Bronwyn Davidson, Julie Marshall, Clement Amponsah, Josephine Ohenewa Bampoe

**Affiliations:** 1 Korle Bu Teaching Hospital, ENT Department, PO Box 77, Korle Bu, Accra, Ghana; 2 Faculty of Health Sciences, University of Sydney, 75 East Street, Lidcombe, NSW 2141 Australia; 3 Department of Audiology & Speech Pathology, The University of Melbourne, 550 Swanston Street, Melbourne, VIC 3010 Australia; 4 Health Professions Department, Manchester Metropolitan University, 53 Bonsall Street, Manchester, M15 6GX UK; 5 Department of Audiology, Speech and Language Therapy, School of Biomedical and Allied Health Sciences, University of Ghana, PO Box 143, Korle Bu, Accra, Ghana

**Keywords:** Help-seeking, Self-help, Communication disability, Speech-language pathology, Sub-Saharan Africa, Community, Ghana

## Abstract

**Background:**

In low and middle-income countries, such as Ghana, communication disability is poorly recognised and rehabilitation services for people with communication disability are limited. As rehabilitation services for communication disability develop, and the profession of speech-language pathology grows, it is important to consider how services can most appropriately respond to the needs and preferences of the community. Understanding the ways in which people currently self-help and seek help for communication disability is central to developing services that build on existing local practices and are relevant to the community.

**Methods:**

A qualitative descriptive survey was used to explore likely self-help and help-seeking behaviours for communication disability, in Accra, Ghana. The survey required participants to describe responses to hypothetical scenarios related to communication disability. A mix of theoretical sampling and convenience sampling was used. Qualitative content analysis was used to analyse data and develop categories and subcategories of reported self-help behaviours and sources of help and advice for communication disability.

**Results:**

One hundred and thirty-six participants completed the survey. Results indicated that community members would be likely to engage in a variety self-help strategies in response to communication disability. These included working directly with a person with a communication disability to attempt to remediate a communication impairment, altering physical and communication environments, changing attitudes or care practices, educating themselves about the communication disability, providing resources, and responding in spiritual ways. Participants indicated that they would seek help for communication disability across a range of sectors – including the Western healthcare, religious, and traditional sectors.

**Conclusions:**

Understanding existing community actions to self-help and help-seek may allow emerging communication rehabilitation services, including the profession of speech-language pathology, to build on existing community practices in resource-limited contexts such as Ghana.

## Background

Responses to illness and disability are diverse and shaped by a complex interplay of culture, community context and personal factors. The ways in which people respond to illness and disability may impact their long-term outcomes [1] as these responses influence decisions about intervention. Responses may include how families react to disability, including how they self-help, and where they seek help for the person with a disability. An understanding of what people do to help themselves and their families, and whether and when people choose to access particular services, is useful in planning and adapting rehabilitation services to make them relevant to local contexts.

The development of responsive and relevant rehabilitation services is particularly crucial in low and middle-income countries^1^ (LMICs), including those of sub-Saharan Africa (SSA) where many people with disabilities (PWD) continue to lack access to basic rehabilitation services [2–5]. Scaling up rehabilitation is on the global agenda, with recent calls by the World Health Organization for countries to address severe rehabilitation workforce shortages, and improve service co-ordination, funding and facilities [6, 7]. In response to the World Report on Disability [8], the World Health Organization’s ‘Global Disability Action Plan 2014–2021’ targets improvements to rehabilitation services and supports for people with disabilities [7]. The recent high-level meeting, ‘Rehabilitation 2030 - A Call for Action’, brought together stakeholders from across the globe to discuss the ways forward, and included a commitment by participants to work towards developing the rehabilitation workforce relevant to each country context [9]. Workforce shortages, and challenges in achieving an appropriate skill-mix amongst rehabilitation workers, are well documented barriers to the development of appropriate rehabilitation services [10, 11].

Rehabilitation services for people with communication disabilities (PWCD) are particularly challenging in SSA [12–14], despite broad estimates suggesting that between 25 and 49% of PWD may experience some form of communication disability^2^ [15, 16]. In SSA, community-based rehabilitation (CBR) workers frequently lack training in communication disability [8, 17]. The availability of speech-language pathologists^3^ (SLPs), professionals who provide communication disability rehabilitation in High Income Countries^1^ (HIC), has been virtually non-existent, with many countries across SSA reporting few, if any, SLPs in-country [18, 19].

Wickenden outlined a range of conditions that may indicate readiness for the development of communication disability rehabilitation services in LMICs, including the development of the profession of speech-language pathology (SLP) [20]. These included a range of indicators related to political stability, economic development, and governance; development indicators – including health, education, mortality, and morbidity outcomes; implementation of key human-rights conventions; growing disability awareness and activism; and the existence of a range of specialized skills and services in related fields. Ghana is a lower middle-income country [21], well-placed to develop rehabilitation services for people with communication disability. It is a well-recognised leader in West Africa, in key areas including economic development and freedom of speech [22]. It has a policy of universal primary education [23], has demonstrated steady improvements in health care [24] and is a signatory to the Convention on the Rights of Persons with Disabilities [25]. Significant challenges remain in the organisation and delivery of rehabilitation services [26, 27]. Both health-related rehabilitation services and CBR approaches have been adopted in Ghana [27], although both approaches continue to be significantly underdeveloped, with concerns about poor uptake of CBR services by PWD, and a paucity of professionals, including physiotherapists, occupational therapists and SLPs, to provide health-related rehabilitation [28]. The SLP workforce in Ghana reflects the wider workforce challenges across SSA [18] with estimates of approximately five practising SLPs [29, 30] providing services in a country of 27 million people [31]. During the past decade, training for SLP has commenced in a number of sub-Saharan African countries [14], including Ghana [30].

While development of the SLP profession is a laudable goal in increasing rehabilitation services for PWCD in SSA, it requires critical examination [32]. SLP is a profession based on Eurocentric cultural beliefs and practices [32, 33] Eurocentrism emphasizes cultural beliefs and values originating from European or “western” cultures, often to the exclusion of other worldviews [33]. Eurocentric beliefs are reflected in the approaches to rehabilitation used by the SLP profession. For example, Western parenting approaches including parent-child style of play are often used in  SLP. Geiger [34] highlighted the need for communication rehabilitation to adopt culturally-specific approaches in the African context, building on existing and culturally relevant resources and practices.

In Ghana, as in much of SSA, Eurocentric approaches to the management of disease and disability have been adopted, however traditional beliefs and the use of traditional interventions also continue to commonplace [35–37]. Spiritual beliefs regarding disability causation and treatment are widespread in SSA, including Ghana [29, 37, 38] with a number of traditional responses being linked historically to neglect, abuse, and infanticide [39–41], however a mix of beliefs is likely to be present in the community, influenced by a variety of factors including the adoption of religion and Western lifestyles [29, 42].

Traditional interventions and religion appear to play a key role in the lives of people with disability in SSA [38, 43]. Kassah [44] described the situation for PWD in Ghana, where “the shrines of traditional healers and spiritual churches become the abodes of the stigmatized” (p 70), as belief in the potential for cure of a number of disabilities, including intellectual disability, remains strong [29]. The desire for curative solutions to disability is unsurprising as, despite growing awareness of the rights of PWD in Ghana [42, 45], stigma associated with disability continues to be widespread [44, 45].

There is limited research exploring community responses to communication disability in the region. Outside SSA, Hopf et al. [46] replicated and expanded the method described in this paper, undertaking a community survey of 144 people in Fiji. They found that community members indicated that they would seek information, assess the PWCD, teach new skills, pray and attempt to change behaviours of themselves and others, in response to the communication disability. Community members would reportedly seek help from a range of sources, including both western medicine and traditional services. Within SSA, Semela [43] described the services that faith healers provide for families in South Africa and discussed the importance of culture and traditional belief in the provision of rehabilitation services for communication disability. This has been supported by more recent research describing the importance of both belief and tradition in service provision for PWD/PWCD [38, 47].

In Ghana, it is not known how, or if, people who experience communication disability and their families attempt to help themselves or seek help. This exploratory study aimed to describe the variety of self-help actions community members in Accra would undertake, and to examine the range of sectors or professions that community members in Accra would use to seek help, if they experienced communication disability within their family.

## Methods

### Study design

This study used qualitative description [48] to explore the range of likely self-help and help-seeking behaviours of community members in Accra. Qualitative description is valuable in social and behavioural sciences research which aims to explore and describe situations and focus on the “what rather than how or why something has happened” ([49], p 129). This research is located within a pragmatic research paradigm, which asserts that interaction between belief and action creates meaning and shapes the search for knowledge [50]. Methodology selection in pragmatic research is guided by the potential use or consequence of the outcome of the enquiry [51].

A qualitative, oral survey was used to gather a range of perspectives from a diverse group of community members. Qualitative surveys are useful in exploring the diversity of experiences within a population or community of interest [52, 53]. Qualitative surveys with community members were selected over other possible research methods, such as in-depth interviewing, as gathering a range of perspectives was an aim of this early exploratory research.

### Materials

Based on the experiences of the researchers working as SLPs in Accra, two hypothetical scenarios and nine open questions relating to the scenarios were developed (Appendix). The survey asked participants to consider how they would respond to each scenario if it occurred within their family. The first scenario described a child with delayed language of indeterminate cause. The subsequent scenario described an adult with a sudden deterioration in speech and associated facial droop. The survey also included basic demographic information. Surveys were conducted orally to accommodate potential variability in literacy and language use within the community.

To address the potential for social desirability bias [54] and sensitivities around seeking help in traditional sectors, indirect questioning was used. Indirect questioning is a technique which asks participants to report on what others may do or say [55]. Direct questioning asks participants to report on their own likely actions, for example, “What would you do”? In contrast, indirect questioning asks participants to report on the perceived likely actions of others, for example, “What do you think other people you know may do?”.

This paper reports on responses to seven survey questions related to help-seeking and self-help. These are listed in the Appendix as Q 1–3,5–7, and 9. A future paper will focus on what service providers might do for communication disability, and will report on data from questions 4 and 8 of the survey.

### Setting

Surveys were all conducted within the Greater Accra metropolitan area within the period September to December 2014.

### Sampling and recruitment

Two types of sampling were applied in order to maximize sample diversity. Theoretical sampling [56] was used to select sites frequented by participants with particular characteristics, with respect to age, gender, education and cultural background. Data were collected at four locations in the Greater Accra region: a tro-tro or minibus station; a roadside restaurant, known locally as a chop bar; a church and a shopping area adjacent to a market. At each site, convenience sampling [57] was employed to recruit members from the community. For inclusion, participants were required to be aged 18 years or older, resident in Ghana and able to give voluntary, informed consent.

### Data collection procedure

Information about the research was provided to community members in each location. At the shopping area and tro-tro station, a team member provided verbal information to passers-by within the vicinity of the data collection point. At the church and chop bar, information about the research was provided as part of the regular service. Interested individuals proceeded to the data collection point. At the data collection point, researchers provided further information, co-read the participant information statement in the participant’s preferred language, and obtained verbal consent. Questions were read out verbatim from the survey instrument. Surveys were offered in three languages [Twi, Ga and English], which are widely spoken in Accra [58]. Responses were translated into English and transcribed contemporaneously by the interviewers.

Surveys were conducted by two Ghanaian team members, of Ga and Larteh ethnic origins respectively, and one Australian team member of European descent, who was resident in Ghana. Each survey took approximately 10 min to complete.

Data collection at each site continued for 3–4 h. Following data collection at each location, the research team undertook an informal review of the range of responses and the mix of participant characteristics. This aided decision-making regarding the need for, and location of, subsequent data collection, for example choosing a site which may be frequented by more women.

### Data analysis

Qualitative content analysis [59] was used to analyse responses. Child and adult-scenario responses were considered separately. Responses to direct and indirect questions to each scenario were collated and analysed together. To indicate if the technique of indirect questioning for the potentially sensitive issue of help-seeking was effective in increasing the variety of responses, responses to direct questions only were subsequently compared with the combined dataset. [Note: Responses from indirect questioning could not be treated separately, as the indirect questioning technique within the interview was used as an adjunct to responses already given to the direct question.]

Initial coding categories were developed by the research team, derived from everyday knowledge, based on experience of working with PWCD in Ghana. Categories were further refined and subcategories developed inductively through careful and repetitive reading of the data. Subcategories were reviewed through a process of subsumption, where the first author reviewed the data item by item, and determined if it fitted into the existing codes, or required an alternative code to be developed [59]. To maximise the rigour of the coding, all data were coded by the first author, with 33% of data independently coded by the two Ghanaian researchers. Differences in coding were discussed and resolved by mutual agreement. Data are displayed by code, with examples for each subcategory. Absolute frequencies are included for descriptive purposes to identify the concepts most frequently reported by participants [59, 60]. Demographic data were explored to consider sample diversity.

### Trustworthiness

Consistent, with the principles of rigour in qualitative research [56], a range of strategies were employed to maximize trustworthiness, including clearly describing the context, participants, data collection and coding processes, use of multiple coders, immersion in data during analysis, use of an expert panel in coding decisions and use of illustrative examples to provide transparency to coding decisions [59, 60]. The panel for coding decisions in this study consisted of a mix of researchers familiar with the context, as well as researchers with a breadth of experience in qualitative coding.

## Results

### Sample demographics

One hundred and thirty-six participants completed surveys across four sites (church *n* = 41, chop bar *n* = 25, bus station *n* = 38, market area *n* = 32). One person commenced the survey but elected to withdraw during data collection. The majority of participants were male (58%, *n* = 79). Participants reported speaking 18 different main home languages. The largest proportion of participants (39%, *n* = 53) reported an Akan language as their main home language (Twi, 32.4%, *n* = 44; Fante 6.6% *n* = 9). Within this sample, just over 20 % (21.3%, *n* = 29) of participants indicated that Ga was their principal home language. Other home languages frequently reported included Ewe (13.2%, *n* = 18) and Hausa (8.8%, *n* = 12). One respondent (0.7%) did not respond to this question. Fifty two percent of participants elected to complete the survey in English (*n* = 71) with the remainder completing their survey in Twi (*n* = 47, 35%) and Ga (*n* = 18, 13.2%). Age and educational attainment profiles of participants are outlined in Table 1, and compared to census data [58] for Greater Accra.

**Table 1:** Age and educational attainment of participants, with comparisons to 2010 census data for Greater Accra [58]

Age Group								
	18–19 years	20–29 years	30–39 years	40–49 years	50–59 years	60–69 years	70 years +	Unspecified
Sample Data	2.2% (*n* = 3)	29.4% (*n* = 40)	33.8% (*n* = 46)	16.2% (*n* = 22)	11.0% (*n* = 15)	5.1% (*n* = 7)	1.5% (*n* = 2)	0.7% (*n* = 1)
Census Data	6.4%	35.6%	25.3%	15.3%	9.0%	4.6%	3.8%	0.0%
Highest educational attainment								
	Did not complete primary education	Primary education	Middle School or JHS	Secondary School or SHS	Vocational, Technical	Other Post-Secondary	Bachelor’s Degree or Higher	Unspecified
Sample Data	7.1% (*n* = 10)	0.7% (*n* = 1)	27.2% (*n* = 37)	17.6% (*n* = 24)	13.2% (*n* = 18)	2.2% (*n* = 3)	31.6% (*n* = 43)	0.0% (0)
Census Data	11.5%	10.0%	37.9%	20.9%	5.2%	7.5%	7.0%	0.0%

### Self-help actions

Participants were asked what they, or other people, would be likely to do, or change, at home to help a relative with a communication disability. When responses to the child and adult-scenarios were collated, fifteen subcategories of responses clustered into seven over-arching categories.

#### Environmental responses

Participants reported they would be likely to make changes to the environment of the person with a communication disability, including both the physical and communication environments. Responses in this category included both changes to the physical environment and changes to the communication environment including altering interaction styles, and the use of specific communication strategies, such as sign language.

#### Skills development and curative responses

Activities focussed on skill development or remediation of difficulties were described by participants as a likely form of self-help in response to communication disability. This category of responses included activities which aimed to effect change within the individual. Types of responses in this category included teaching specific skills, use of herbal remedies, first aid and exercise.

#### Attitudinal responses

Participants indicated that attitudes in response to communication disability may impact the way they engage with PWCD. Both positive and negative changes were described, including being more patient, avoiding contact with the individual, and pressuring the person with a communication disability to speak. Nine participants described a likely sense of helplessness.

#### Further learning responses

Participants also indicated that they may attempt to learn more about the communication disability, by examining or assessing the person themselves, or educating themselves about communication disability.

#### Resources responses

Providing resources, such as money and learning resources for the PWCD were also described as likely means of self-help by participants.

#### Spiritual responses

Spiritual responses, including fasting and prayer were described by participants as a likely means to self-help in response to communication disability.

#### Care responses

Increasing care for the PWCD was also described by respondents as a likely change if they were to experience communication disability in their family.

Categories and subcategories of responses, with illustrative examples, are provided in Table 2.

**Table 2:** Categories, subcategories and examples of self-help activities from child scenario (C) and *adult scenario (italicized) (A)*

Category (Total response count for category)	Subcategory	Examples and number of responses in subcategory	Response Counts	
			Child	Adult
Environmental (Child scenario, 61) (Adult Scenario, 3)	Interaction	Try talking to him, asking him questions, following what he comes up with. (C)	30	1
		I will see if she/he will respond to a touch or throw something away and see if the child will talk or not, to prompt the child. (C)		
		*If age related, I will interact more with him. (A)*		
	Environment	Send the child to school. As the child mingles with other children he will learn from them. (C)	6	0
		…changing the environment. Taking the child to a place where there are colours, pictures, toys, things that kids would like to play with to see and touch. (C)		
	Communication strategies	Make signs (participant gestures with hands) and see if the child will respond. (C)	25	1
		Sign language; using audio-visual means It will help it stick in the person’s mind. (C)		
	Correction	*When I am at home I will correct his speech. (A)*	0	1
Skills development / curative (Child scenario, 26) (Adult Scenario, 8)	Specific teaching	I will use pictures also to teach the child. (C) Use sound and what the child likes to teach him. Make sounds and see if the child will imitate. (C)	24	0
	Home herbal remedy	I may find out herbs that may be helpful and prepare it. (C) *I will try herbs or pray (A)*	2	4
	First aid	*If I know of any first aid I will do it* e.g. *Pouring water on him or her (A)* *There is a first aid given for such ones. I will quickly find it and give him. (A)*	0	3
	Exercise	*I will also encourage the person to exercise. (A)*	0	1
Attitudinal (Child scenario, 25) (Adult Scenario, 0)	Attitude	More patience is needed first to handle such a person, to concentrate on him/her. (C)	12	0
		I would not associate myself to her like my other children who talk. (C)		
	Helplessness	I don’t think I can do anything. I don’t have any skill to help with talking. (C)	9	0
		I don’t have any help for that child. (C)		
	Pressure to speak	Force him to talk so he will try to talk. (C) Every day we must force him to speak as it is our future. (C)	4	0
Further learning (Child scenario, 13) (Adult Scenario, 0)	Observation and assessment	If I have another child or there is a child of the same age, I’ll call the child, look through his mouth and see if there are differences. (C) Observe them, study them, discuss with my wife. (C)	11	0
	Self-education	I would learn about how to help, in that I buy books, read. (C)	2	0
Spiritual (Child scenario, 12) (Adult Scenario, 8)	Spiritual actions	Fasting and prayers (C) *I’ll be praying or asking intercessors to help me with prayers (A)*	12	8
Resources (Child scenario, 11) (Adult Scenario, 3)	Funding and resources	I will provide food for the child. I will also provide money for school. (C) *I will give the person some money (A)*	6	1
	Educational aids	Provide materials that could help the child to talk - a state of the art aid to help the child to talk. (C) Looking for reading aids e.g. ABC chart (C)	4	0
	Employ others	Employ the services of a teacher who will help with the talking. (C) *I will employ a teacher to help him (A)*	1	2
Care (Child scenario, 2) (Adult Scenario, 2)	Care given	Will pamper her. Make her happy always. (C) *We need to pamper him. (A)*	2	2

Frequency of responses within categories and subcategories are included

### Help-seeking for communication disability

Participants indicated that they would likely seek help from a range of sources in response to both child and adult-scenarios, with participants frequently describing more than one source of help.

Help-seeking for both the adult and child-scenarios was collated into five categories representing the service sectors used for help seeking: western healthcare; religious; traditional belief; community and education sectors. The remaining responses (*n* = 6) represented a heterogeneous range of sources of help, included as “other”. People and places identified in each sector, for both child (C) and adult (A) scenarios are reported in Table 3, with absolute frequencies, the number of people who mentioned that person or place, provided to illustrate the patterns within each category.

**Table 3:** Help-seeking for communication disability, by sector (category) and type of person/place (subcategory)

Western healthcare sector			Religious sector			Traditional belief sector		
	C	A		C	A		C	A
Doctor	94	115	Pastor, priest	29	23	Herbalist	41	34
Hospital	19	8	Church	20	22	Spiritualist	24	23
Medical specialist	12	3	Prayer camp	6	8	Fetish priest^b^	17	11
Other professional	8	6	God	4	5	Traditional doctor (type unspecified)	0	13
Nurse	5	5	Mallam^a^	2	4	Native doctors	7	4
SLP / speech specialist	5	0	Imam	1	0	Shrine	4	2
Midwife	1	1	Religious councils	1	0	Witch camp^c^	1	2
Speech and hearing centre	1	0				Witchdoctor	2	0
Health centre	1	0				To the river^d^	1	0
Psychiatric hospital	0	1						
*Total*	*146*	*139*	*Total*	*63*	*62*	*Total*	*97*	*89*
Community sector			Education sector			Other response types		
Community members	16	11	School for the deaf	16	0	Parliamentarian	1	0
Elderly person	12	4	Education/school (general)	10	0	Non-government organisation	1	1
Orphanage	1	0	Teacher	6	2	Gym	0	1
			Special school	3	0	Government	0	1
						Asylum	0	1
*Total*	*29*	*15*	*Total*	*35*	*2*	*Total*	*2*	*4*

All responses (direct and indirect questioning), with absolute frequency counts, C = child case scenario, A = adult case scenario. [Participants could report that they would seek help from more than one type of person in each sector, e.g. doctor, nurse]

^a^ an Islamic scholar

^b^ a traditional priest communicating with the spirit world who typically resides at a shrine

^c^ a community reserved for those thought to be witches

^d^ refers to a traditional spiritual practice of leaving children with disabilities or deformities “by the river”

A diverse range of people and places associated with the western healthcare sector were identified as sources of help in response to both the adult and child scenarios, with doctor the most commonly reported source of help. Help-seeking in the religious sector was also a frequent response to the scenarios, with pastors, priests and churches the most frequently identified source of help within the religious sector. Within the traditional medicine sector, a variety of help sources were identified, with herbalists^4^ and spiritualists named frequently as sources of help. Sources of help in the community included elderly community members, other community members such as family and friends, and people at the orphanage. Education sector responses were predominantly in response to the child-scenario, and included a mix of general and special education resources.

### Comparison of direct and indirect responses

No additional data on self-help were generated in response to the indirect questions. Direct question responses and combined (direct and indirect) responses are compared in this section (Table 4).

**Table 4:** Sector ranking for help-seeking – child and adult scenarios. The influence of direct and indirect questioning

Child scenario					Adult scenario				
Direct Responses only (n)		RANK	All Responses - direct + indirect, % (n)		Direct Responses only (n)		RANK	All Responses - direct + indirect, % (n)	
60% (130)	Western healthcare	1	Western healthcare	39% (146)	80% (133)	Western healthcare	1	Western healthcare	45% (139)
13% (29)	Education	2	Traditional belief	26% (97)	8% (14)	Religion	2	Traditional belief	28% (89)
10% (21)	Community	3	Religion	17% (63)	6% (10)	Traditional belief	3	Religion	20% (62)
9% (20)	Religion	4	Education	9% (35)	4% (7)	Community	4	Community	5% (15)
7% (16)	Traditional belief	5	Community	8% (29)	1% (1)	Education	5	Other	1% (4)
0% (0)	Other	6	Other	<1% (2)	<1% (1)	Other	6	Education	<1% (1)

Sector (%) as a proportion of total responses

When direct questioning was used, the Western healthcare sector was most frequently for help-seeking for both child and adult scenarios. When both direct and indirect responses were combined, Western healthcare settings remained the most commonly identified sector for help-seeking, however the proportion of responses attributed to the Western healthcare was smaller (see Table 4). In the child scenario when direct responses only were considered, 60% of the total number of responses indicated seeking help in western healthcare settings. When direct and indirect responses were combined, the Western healthcare sector accounted for only 39% of responses. A similar pattern was evident in the adult scenario where proportions of 80% (direct questioning) and 45% (combined data) indicated seeking help in Western healthcare settings.

Two sectors were consistently reported more frequently when indirect questioning responses were included in the data. For the child scenario, seeking help in the religious sector rose from 9% on direct questioning to 17% when indirect responses were included. A similar rise was evident in the proportion of adult scenario respondents indicating help-seeking within the religious sector, from 8% for direct questioning responses only, to 20% when responses to direct and indirect questioning were combined. Consulting within the traditional belief sector also demonstrated increased frequency when indirect responses were included (7% to 26% for the child scenario, and 6% to 28% for the adult scenario). Rankings and comparison of direct questioning responses and combined direct and indirect questioning responses regarding help-seeking for both adult and child scenarios are reported in Table 4.

## Discussion

This qualitative inquiry explored reported self-help and help-seeking behaviours in response to communication disability in Greater Accra, Ghana. It aimed to include a diverse range of participants from across Greater Accra to ensure that a range of community perspectives were considered. Participants were from a range of age groups, broadly consistent with the 2010 census data for Greater Accra [58]. Both men and women participated in the survey, however there were slightly more male respondents, with 58% of male respondents as compared with the 52% male population identified within Greater Accra [58]. This may be linked to site selection or to a variety of socio-cultural factors, such as education, confidence or familiarity with surveys. Twi and Ga language speakers were dominant in the sample, consistent with census data indicating that 39.7% and 27.4% of Greater Accra residents identify as Akan or Ga ethnicities [58]. Inclusion of participants with a wide range of other Ghanaian home languages including Hausa and Ewe indicated ethnic diversity amongst participants. There was a higher proportion of university educated participants, when compared to the general population [58], which may be related to sites selected or as a result of community members with higher education being more comfortable participating in surveys.

### Self help

Results from this study suggest that the community members surveyed would make active attempts at intervention, using a variety of approaches, to assist a person experiencing communication difficulty in their family. This is consistent with Fijian research indicating that people would be likely to attempt a range of self-help actions consistent with their culture [46]. Previous research from HICs indicates that parents of children with communication disabilities who are aware of issues may take action themselves [61] and/or undertake a period of waiting, prior to seeking professional help [62].

Within this study, participants described self-help actions which aimed to effect change within the individual with a communication disability, using specific teaching, herbal remedies, first aid and exercise. Research in other countries also found that parents of children with a communication delay may use direct teaching and imitation, prior to seeking help [46, 61]. However, the use and acceptance of direct teaching of language and communication skills appears to be influenced by culture [63, 64] and requires further exploration in the Ghanaian context.

Participants also described changing the physical and communication environments in response to a communication disability. This is also consistent with both results from the Fijian study [46] and previous UK-based research, indicating that both SLPs and families believed that the environment plays a role in language development [61]. SLP interventions frequently target the communication environment using a range of approaches. These may include changing interaction opportunities, such as asking questions, and interacting more or less; altering the communicative environment, for example sending the child to school or to use of more stimulating environments; and changing the communication style, for example using gesture or visual cues [65].

The International Classification of Functioning, Disability and Health (ICF) [66] outlines a conceptual framework for the biopsychosocial model of disability, with five dimensions interacting to influence human functioning [67]. Community members in this study indicated they would be likely to use self-help strategies, which focus on both changes within the individual and the environment. Self-help strategies described in this study, which focus on effecting change within the individual, such as direct teaching, are consistent with the ‘body structure and function’ component of the ICF [66], whilst environmental responses are consistent with the ‘environment’ component of the ICF [66]. The range of approaches to self-help, described by participants, reflect the diverse influences contributing to communicative functioning and are consistent with the types of interventions used by SLPs [67].

Importantly, if both direct teaching and environmental interventions are part of current self-help responses to communication disability in Accra, then families may be receptive to, and reassured by, information about ways to enhance their environmental and direct teaching interventions, even before they formally seek help. Community members in this research indicated a desire to both observe and understand their family member’s communication disability or to seek information if their family member was affected, raising the need for accessible community level information on communication disability. Public awareness of communication disability is frequently limited in LMICs [12, 15, 20] despite evidence of the effectiveness of early intervention for a number of developmental communication delays [68, 69]. SLPs in LMICs are well-placed to consider how best to support self-help actions taken by families, through provision of community-level education and engagement [70, 71].

Typically, SLP services in HICs have adopted approaches which focus on intervention targeting the needs of individuals [71, 72]. Involvement of SLPs in approaches that develop community-wide education and capacity-building, described above, would suggest a shift in focus for SLP services to a form of public health SLP [71, 72] with the emphasis on the early intervention efforts of family and other community members. Such community level interventions may be particularly important in LMICs, such as Ghana, where rehabilitation services are extremely limited [26, 27]. Simply increasing the rehabilitation workforce to provide individualized clinical services, in the form of SLPs or other workers addressing communication disability, will be ineffective in meeting population needs for many years [19].

Community members indicated that responses to communication disability may include care, nurturing and a range of attitudinal responses. This is consistent with responses, described as behavioural, in Fijian research [46]. Development of positive attitudinal responses to communication disability are important as communication disability has been associated with stigma in Ghana [73]. Communication disability rehabilitation services, including SLPs, in LMICs are likely to need to adopt broader roles in supporting PWCD than their counterparts in HICs, which may include awareness raising and activism [20].

Spiritual activities were described as likely responses to communication disability. Data from this study reveal that community members are likely to respond to communication disability through spiritual activities, including prayer and fasting. Whilst this study did not explore whether the spiritual actions were aimed at cure, or coping, further understanding of how community members use spiritual activities to help-themselves could guide the development of appropriate supports in this area.

Extending this preliminary research, through detailed exploration of self-help activities would be beneficial, in order to begin to build communication disability rehabilitation practices which harness, support and extend community knowledge and practices [34, 46] and allow the emergence of a more Afrocentric approach to communication disability rehabilitation [74, 75].

### Seeking help for communication disability

Cultural beliefs, including religion, spiritual beliefs and societal norms, influence how people respond to health conditions and seek help for them [38, 43, 76]. This research suggests that participants would seek help for communication disability across a variety of sectors in Accra, many of which sit outside the Western biomedical paradigms. Results of this study indicate that help-seeking behaviours are consistent with the understanding that disability in Ghana is viewed as multidimensional, with a blending of traditional, religious and Western belief [44]. Knowing which sectors people may access for communication disability is important, as trust between clients and service providers has been shown to be an important aspect of accepting advice, complying with treatment and achieving behaviour changes necessary for good health [75, 76]. Siminoff [77] stressed that “the experience of illness, help-seeking and treatment is not just a biological but also a social process” (p5) and involves relationship building between service users and providers.

Comparing responses when participants were asked directly about their own likely help-seeking behaviours, and help-seeking behaviours they believed others may engage in, indicates the possibility that perceptions around social acceptability may affect responses related to sharing information about help-seeking [54], and should be addressed sensitively in research in this field.

Results of this research suggest that, in the case of communication disability in Accra, trusted service providers for PWCD are situated in a variety of sectors. For example, if an individual’s first response is to seek advice from their pastor, they may be likely to trust information given by that person, reflecting their cultural beliefs about causation and trajectories of communication disability.

In HICs where health-related rehabilitation services are more readily available, multidisciplinary approaches to rehabilitation are often promoted [78]. The range of professions inferred in the term ‘multidisciplinary’ is often linked to the Western healthcare sector, including doctors, audiologists and occupational therapists [78, 79]. In Ghana, an even broader approach to rehabilitation is indicated, involving collaboration across sectors, including Western healthcare, religion and traditional belief. Furthermore, acknowledgement of a more inclusive team across sectors is worthy of consideration in increasingly diverse communities in HICs. As communication disability rehabilitation services develop in Ghana, creating links and establishing dialogue about communication disability across sectors currently accessed by the community may be an appropriate way forward to ensure emerging services fit within the existing service landscape [80].

Differing beliefs about causation of communication disability may make multisectoral approaches challenging. However, increasing dialogue and collaboration, between SLPs, religious leaders, traditional practitioners, education professionals and community leaders around communication disability, may ultimately mean that families have access to information and support, from the people they trust.

### Limitations

This exploratory study provides preliminary information from a sample of participants in one Ghanaian city, and must be interpreted conservatively. Also, it is of note that brief qualitative surveys do not allow deeper exploration of responses.

Participants came from a cross section of the community however this study did not aim to be representative of the population of Accra, or Ghana. The characteristics of the sample may have influenced the content of responses, for example, strong representation of respondents with a university level education. Some groups, not necessarily identifiable from census data, may not have been accessed, for example, parents of children with disabilities, people from very impoverished backgrounds. The use of the two specific hypothetical scenarios may not have elicited all activities that would be undertaken by participants in relation to children and adults with a communication disability. Responses may have been influenced by respondent’s desire for social acceptability.

In an effort consider sample diversity, home language data were treated as a proxy for ethnicity data from the national census [58], despite home language representing one possible component of ethnicity. In a short survey, it is not possible to ascertain participants’ understanding, or experience with, communication disability, which may have influenced their responses.

## Conclusion

Despite limited recognized rehabilitation services for communication disability in Ghana, this descriptive study of help-seeking and self-help for communication disability indicates that people would be likely to undertake a range of self-help actions and seek help across a variety of sectors if they experienced communication disability within their family. Developing a knowledge base regarding the type of self-help and help-seeking activities that families are likely to engage in, offers the emerging profession of SLP in Ghana an opportunity to build on the actions already undertaken by families in this resource limited context, and to engage with other sectors from whom families would seek help. A broader view of communication disability rehabilitation, beyond the Western health sphere, may be relevant in the Ghanaian context, to ensure that PWCD have access to a range of services in accordance with both their needs and preferences, and from service providers they trust.

## Appendix

### Oral survey questions

#### Child Scenario

1. I would like you to think about your family. Imagine there was a child in your family who was 5 years old and not yet talking at all. What would YOU do?
2. [probe] Is there anything you think of that you would do yourself, or change at home, to help with the talking? (if not already given)
3. [probe] Who would you go to for advice or help? (if not already given)
4. What do you think that person might do or say?
5. What do you think other people might do, or what are some other places they would go for help?

#### Adult Scenario

6. Imagine that there was an adult in your family who woke up one day and you noticed that their speech was very slurred and difficult to understand. It did not improve. They were also drooling from their mouth a little (use gesture). What would you do?
7. [probe] Who would you go to for advice or help for this type of problem? (if not already given)
8. What do you think that person might do or say?
9. Are there other types of people places that OTHER people might seek help or ask for advice for this type of problem that you haven’t already mentioned?

#### Demographic questions (categorical)

**Table:**

Which ago group are you in?	<20, 20’s, 30’s, 40’s, 50’s, 60’s, 70’s or older
Gender	female / male

What is the highest school level that you have completed?

**Table:**

Never attended formal schooling	Did not complete primary education
Completed primary	Completed middle/JHS
Completed secondary/SSS	Completed commercial / technical
Completed post-secondary (other)	Completed tertiary

## Abbreviations

HICs: High income countries
LMICs: Low and middle-income countries
PWCD: People with communication disabilities
PWD: People with disabilities
SLP: Speech-language pathology
SLPs: Speech-language pathologists
SSA: Sub-Saharan Africa

## Footnotes

1 The term high income countries (HICs), and low and middle-income countries (LMICs) are based on classifications from the World Bank based on GNI per capita [21]. The term HICs is used in this paper to delineate countries that *typically* have more comprehensive rehabilitation services.

2 Communication disability can be defined as a disability where an individual’s ‘…ability to communicate is affected by their response to an impairment and/or social and contextual factors which interrelate with each other and with the person themselves, resulting in impaired communication skills.’ [15, p. 277].

3 Speech-language pathologists may also be called speech and language therapists or speech pathologists in some countries.

4 Whilst herbalists are becoming increasingly accepted as part of medical services in Ghana, within this research they were classified within the ‘traditional belief’ category, due to the traditional origins of the practice [81].
